# BMP signalling in colorectal cancer: losing the yin to WNTs yang

**DOI:** 10.1002/path.6428

**Published:** 2025-04-11

**Authors:** Eloise Clarkson, Annabelle Lewis

**Affiliations:** ^1^ Centre for Genome Engineering and Maintenance, Department of Biosciences, College of Health, Medicine and Life Sciences Brunel University London Uxbridge UK; ^2^ Centre for Genomics and Child Health, Blizzard Institute Queen Mary Univeristy of London London UK

**Keywords:** colorectal cancer, WNT, BMP, BMP antagonists, cancer stem cells, cellular plasticity

## Abstract

Colorectal cancer (CRC) is the third most common form of cancer globally, and arises from the hyperproliferation of epithelial cells in the intestine. The architecture and maintenance of these cells is governed by two major signalling pathways working in a counter‐gradient: the stem cell WNT signalling pathway, and the prodifferentiation bone morphogenetic protein (BMP) pathway. It has long been known that this WNT‐BMP balance is disrupted in CRC, with hyperactive WNT signalling leading to increased proliferation of epithelial cells and tumour progression. BMP signalling, and its prodifferentiation effects, have increasingly become a focus for CRC research. Loss of BMP signalling, and that of its receptors, has been shown to increase WNT signalling and cancer stem cells in CRC. BMP signalling is further modulated through secreted BMP antagonists localised to the intestinal crypts, which create a niche ensuring that sustained WNT signalling can maintain stem‐cell self‐renewal capacity. A number of studies combine to demonstrate the effects of overexpression of these BMP antagonists, showing that hyperactivity of the stem‐cell‐supporting WNT signalling pathway ensues, leading to deregulation of the intestinal epithelium. Cellular hyperproliferation, the emergence of ectopic crypts, and an increase in stem cell numbers and characteristics are common themes, contributing to disrupted epithelial homeostasis, an increase in CRC risk and progression, and resistance to therapy. This review aims to compile the current knowledge on BMP antagonists, their role in CRC development, and how we can utilise this information for biomarker research and novel therapeutics. © 2025 The Author(s). *The Journal of Pathology* published by John Wiley & Sons Ltd on behalf of The Pathological Society of Great Britain and Ireland.

WNT signalling, along with BMP signalling, is the main driving force behind intestinal architecture and is crucial for maintaining homeostasis. The two pathways exist in a finely tuned counter‐gradient, with disruption of proteins in either pathway leading to dramatic changes in the cellular make‐up and function of the intestine.

## Intestinal architecture and the WNT/BMP signalling axis

**Figure 1:**
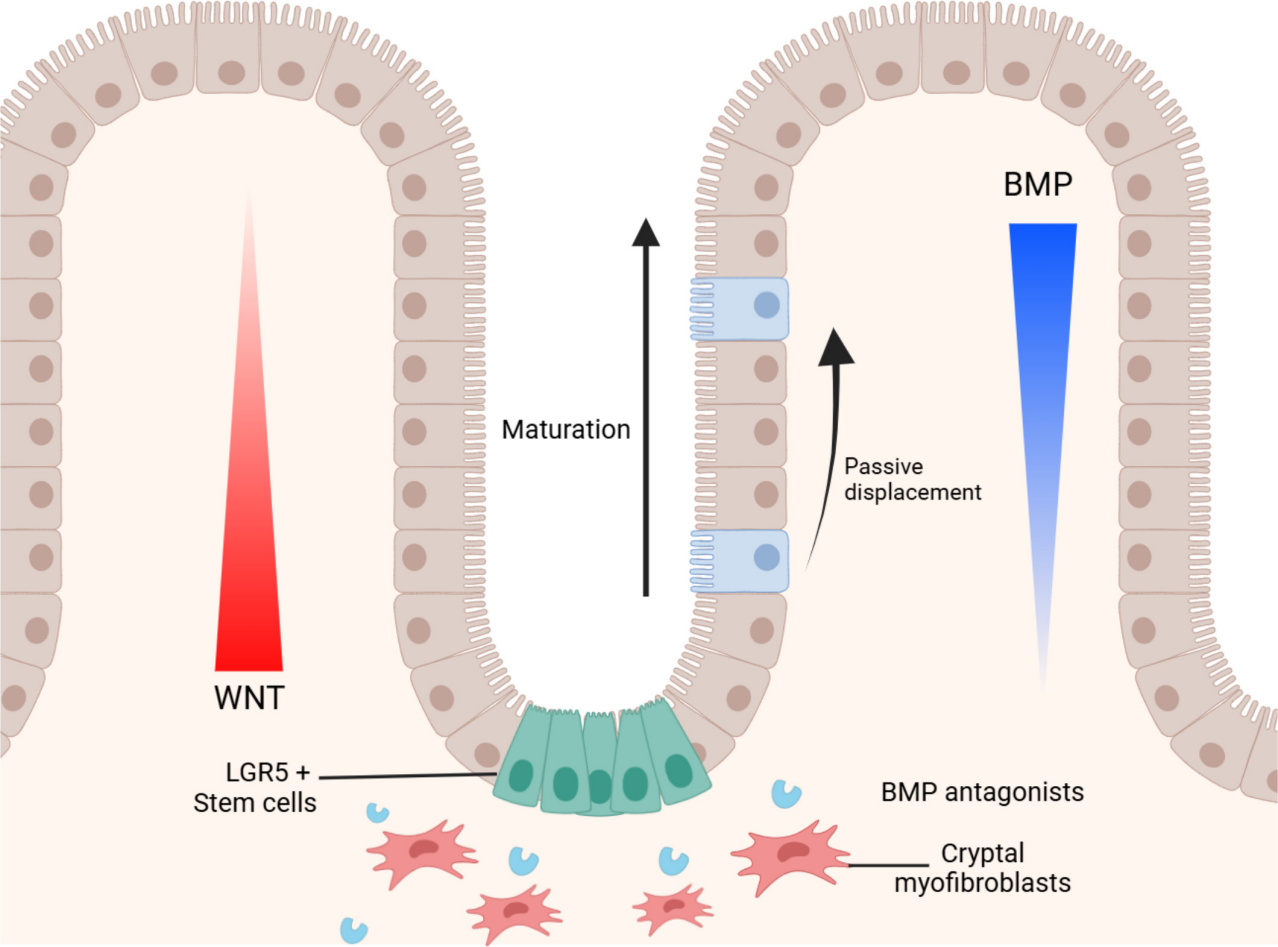
Intestinal epithelial structure with counter‐gradients of WNT and BMP signalling. BMP antagonists are secreted from myofibroblast cells close to the base of the crypts. Created in BioRender. Lewis, A. (2025) https://BioRender.com/e63j988. All terms of the License Terms including all Prohibited Uses are fully complied with.

The intestinal tract is comprised of crypts and villi, with the small intestine characterized by villi protruding into the lumen and crypts invaginating into the mucosa. The large intestine, encompassing the caecum and colon, shares this architecture but lacks prominent villi (Figure 1). Epithelial cells line the intestinal tube and originate from stem cells situated in mitotic zones at the crypt base. These stem cells facilitate the continuous renewal of epithelial cells, which are crucial for maintaining intestinal epithelial integrity.

The WNT signalling pathway is central to the creation and maintenance of the stem‐cell compartment in the crypts. Stem cells within the crypt are capable of self‐renewal, and compete, with neutral dynamics, within their niche to provide a steady production of epithelial cells to maintain the intestinal epithelial lining [1]. They are identified by the presence of LGR5, which is a direct target of WNT signalling.

Bone morphogenetic proteins (BMPs) are a group of cytokines and metabologens that are widely recognised for their important role in morphogenic signalling and are responsible for orchestrating multiple tissue architectures throughout the body. Originally identified through their ability to initiate and direct bone and bone‐cartilage formation [2], they have subsequently been shown to influence a wide range of biological processes, including cancer progression.

**Figure 2:**
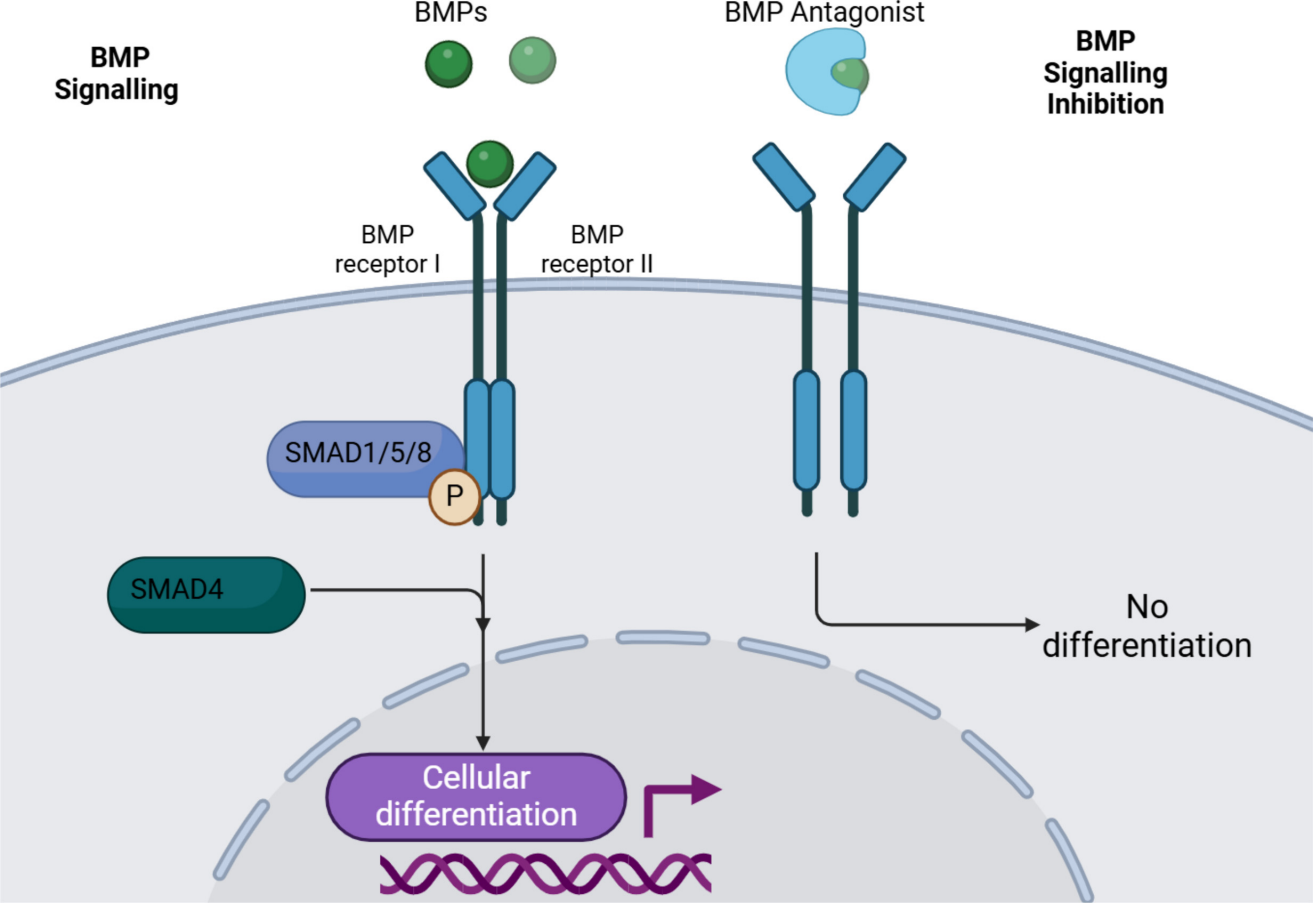
BMP signalling through BMPR I and II by SMAD dependent phosphorylation. Created in BioRender. Lewis, A. (2025) https://BioRender.com/b38k179. All terms of the License Terms including all Prohibited Uses are fully complied with.

BMPs belong to the TGF‐β superfamily, and are secreted as dimers, binding to a complex of transmembrane serine threonine kinase receptors I and II (BMPR I and II) [3]. This initiates phosphorylation of the type I receptor by the type II receptors, triggering phosphorylation of a receptor‐associated SMAD complex (SMAD1/5/8) that subsequently interacts with SMAD4, resulting in translocation to the nucleus to regulate gene transcription [4] (Figure 2). While both epithelial and mesenchymal cells express BMPs and their receptors, BMP antagonists in the intestine are found primarily in the mesenchyme and are expressed by intestinal cryptal myofibroblasts and smooth muscle cells.

Intestinal stem cells, therefore, are acutely sensitive to changes in either WNT or BMP levels, with sustained WNT signalling due to the action of secreted BMP antagonists that block BMP‐induced differentiation [5]. Cells generated from LGR5^+^ cells exit the crypt through passive displacement and migrate along the villi–crypt axis towards the villi tips in a conveyor belt‐like movement. As epithelial cells migrate along the crypt‐villus axis and away from BMP antagonists secreted from the myofibroblasts [6], they are exposed to BMP signalling that promotes differentiation into mature cell lineages before shedding at the villus tip (Figure 1) [1, 6, 7].

## Colorectal cancer

Colorectal cancer (CRC) represents a major global health challenge, ranking as the third most common form of cancer, with 1.5 million new cases annually [8]. Risk factors for CRC include age, smoking, diet, obesity, and physical inactivity, which significantly increase susceptibility [9]. In all, 10–16% of cases are caused by hereditary conditions such as Lynch syndrome and familial adenomatous polyposis [10]. However, the majority of CRCs emerge sporadically through oncogenic mutations in intestinal epithelial cells, particularly affecting the WNT and BMP signalling pathways, which drive hyperproliferation, polyp formation, and ultimately tumour development.

**Figure 3:**
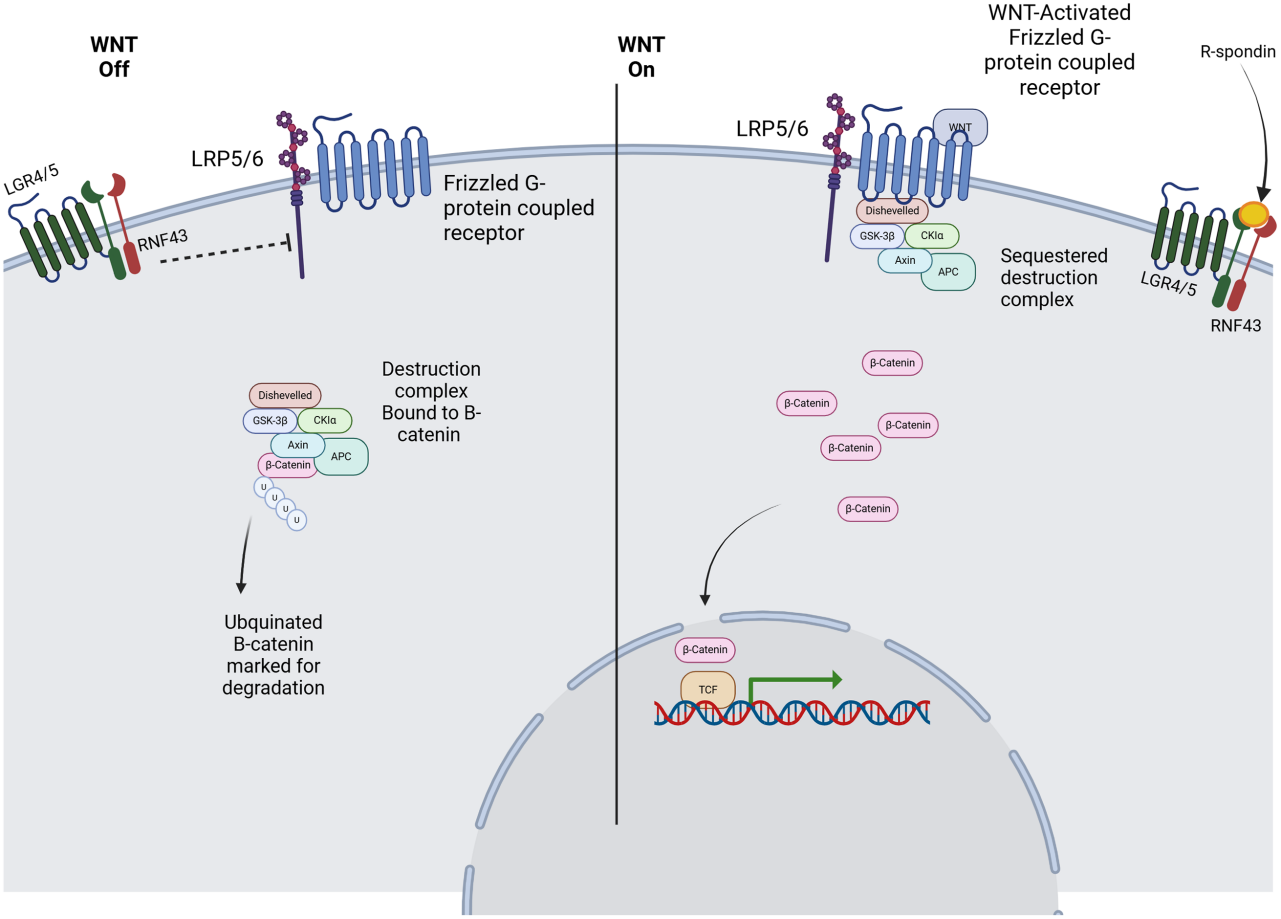
WNT/APC/β‐catenin pathway. WNT binds to G‐protein‐coupled receptors to phosphorylate LRP. Phosphorylated LRP induces translocation of the destruction complex to the membrane receptors, activating Dishevelled, leading to inhibition of the destruction complex. Inhibition of the destruction complex prevents ubiquitination of β‐catenin, leading to an increase of cellular β‐catenin levels. Increased β‐catenin levels lead to increased β‐catenin translocation into the nucleus, which binds to TCF and causes TCF‐mediated transcription of target genes. LRP5/6 is degraded by RNF3/4, but upon binding of R‐spondin to LGR4/5, RNF3/4 is inhibited, allowing WNT activation. The transmembrane receptors LRP5/6 are expressed in the majority of intestinal epithelial cells. However, LGR4/5 are only expressed in undifferentiated stem cells and correlate with increased levels of WNT signalling in these cells [75]. Created in BioRender. Lewis, A. (2025) https://BioRender.com/u23o080. All terms of the License Terms including all Prohibited Uses are fully complied with.

The most well‐understood driver of CRC is oncogenic hyperactivity of the WNT signalling pathway causing aberrant uncontrolled proliferation of epithelial cells [11]. Mutations in the WNT signalling pathway can be inherited or acquired, and most frequently occur within the intestinal stem‐cell compartment of the crypt [12]. The most commonly mutated gene in the WNT signalling pathway is *APC* (Adenomas Polyposis Coli), which encodes a scaffold protein mediating the β‐catenin destruction complex. Mutated APC leads to accumulation of intracellular β‐catenin, which activates proto‐oncogenes (Figure 3) [13], causing cells to exhibit stem‐like properties such as hyperproliferation, increased migration, and resistance to common chemotherapies [14].

In contrast, BMP signalling is the main differentiation pathway of epithelial cells in the villi and loss of BMP signalling due to mutations in a subset of genes in the pathway is often part of initiation and progression of CRC [15]. This complex interplay between WNT overactivation and BMP repression has become more apparent in recent years with identification of the BMP pathway disrupting mutations that underlie inherited CRC syndromes and inherited genetic BMP variants that associate with increased CRC risk [16, 17, 18]. When taken together with well‐known somatically acquired BMP mutations driving CRC progression [19, 20], it is evident that BMP signalling should be considered alongside WNT activation for future CRC therapeutic development.

## BMP signalling in colorectal cancer

BMP signalling in cancer appears to be complex, shown to be both oncogenic and tumour suppressive in nature [21, 22, 23, 24]. Mutations in the intracellular transducers of the BMP signalling pathway are well established in CRC. For example, *MADH4*, which encodes the SMAD4 protein that transduces the BMP signal, has copy number deletions in ~30% of CRC samples [19]. Other reports suggest that ~5–24% of colorectal cancer have inactivating mutations in *SMAD4*, collectively making it one of the most frequently mutated genes in CRC. Alongside SMAD4, the BMP receptor BMPR1A is also frequently lost in juvenile polyposis syndrome, with 17–38% of cases having disease‐causing variants within the gene [20].

### BMP signalling and cancer stem cells

BMP signalling has been shown to influence populations of cancer stem cells (CSCs) in colorectal cancer. CSCs are described as a population of self‐renewing malignant and highly tumorigenic cells that drive tumour initiation and progression. In recent years, our view of the tumour landscape has changed from that of a homogenous pool of dividing cells to a more complex hierarchy, with CSCs giving rise to lineages of cancer cells that form the heterogeneous tumour [25]. CSCs are often termed ‘immortal’ due to their ability to self‐renew and their resistance to conventional chemo‐ and radiotherapies. This makes populations of CSCs able to drive tumour initiation due to their self‐renewal capacity and increases the risk for cancer recurrence by evading therapy [26].

In the intestinal tract, BMPs promote differentiation, apoptosis, and chemosensitization, and are therefore an opposing factor to CSCs. CSCs in the intestinal tract lack BMP expression in comparison with differentiated cancer cells, and addition of BMP4 induces CSC differentiation and chemosensitization [27]. SMAD4 is also instrumental in cellular plasticity, and enforces differentiation and suppresses proliferation driven by oncogenic WNT signalling. Loss of SMAD4 enables dedifferentiation and, subsequently, WNT‐driven hyperproliferation [28]. Furthermore, BMP4 blocks the transplantation ability of CSCs and inhibits tumour growth in the absence of SMAD4 loss [29]. BMP2 was also found to be downregulated in CRC and inhibited cell growth, migration, and other CSC characteristics, as well as enhancing chemosensitivity when overexpressed in colon cancer cells. This was replicated in xenograft experiments where BMP2 overexpression reduced tumour volume [30, 31]. Similarly, BMP3 is downregulated in CRC and this correlates with promoter hypermethylation. Reintroduction of BMP3 in methylated cell lines caused growth suppression [32]. Studies of BMP7 are less conclusive, as high BMP7 correlates with advanced tumour stage and poor prognosis [33]. However, a more recent study found high BMP7 in the earlier stages, but restricted to more differentiated cell populations, suggesting that it can act as a prodifferentiation agent. This was validated by the use of a stabilised variant, vBMP7, which reduced the self‐renewal capacity of CRC stem cells, pushing them towards a more differentiated phenotype and enhancing therapeutic response *in vitro* and *in vivo* [34]. Less is known about BMP9, although its upregulation has been linked with the anticancer properties of tetrandrine [35]. Overall, there are substantial links between elevated levels of BMP, more differentiated CRC cell types, and sensitivity to therapeutics.

A transcriptomics‐based screen for genes required for maintaining the CSC phenotype identified the zinc‐finger transcription factor GATA6 as a key regulator of the WNT and BMP pathways in CRC. It was found that GATA6 directly drove the expression of LGR5 in adenoma stem cells, but restricted BMP signalling to differentiated tumour cells. *Gata6* deletion in mouse colon adenomas increased the levels of BMP factors, thus blocking the self‐renewal capacity of CSCs [36].

## BMP antagonists

It is clear that loss of BMP and hyperactivity of WNT signalling is imperative for cancer progression in the colon. While loss of key BMP transducers is well documented, another modulator of BMP activity is found in the form of BMP antagonists, expressed by mesenchymal cells in the stroma and around the base of the intestinal crypt, and key to maintaining WNT signalling in the stem‐cell niche. BMP antagonists can be categorized into three classes: (i) ligand antagonists, which bind directly to BMPs, (ii) BMP proregions, which complex back with mature BMPs, and (iii) receptor antagonists, which bind to BMPRs to prevent BMP binding to their cognate cell surface receptors [37]. Similar to BMPs, BMP antagonists contain cysteine knots and typically form homo‐ or heterodimers. BMP antagonists can be further broken down into five classes based on this cysteine knot formation: Noggin, the Dan family, the Chordin family, Twisted gastrulation, and Follistatin [38]. Some well‐characterised BMP antagonists include Noggin, which has been implicated in promoting skin and breast cancer tumorigenesis; the Gremlins (encoded by *GREM1* and *GREM2*), with repression of Gremlin1 shown to inhibit tumour cell proliferation; and the Chordin family of proteins, including Chordin (encoded by *CHRD*), Chordin‐like 1 (*CHRDL1*), and Chordin‐like 2 (*CHRDL2*) [39, 40, 41, 42] (Table 1).

**Table 1:** BMP antagonists and associated cancer

BMP antagonist	Cancers identified	Phenotypic display
Noggin	Breast, colorectal, gastric, melanoma.	Proliferation, colony formation, abnormal branching and budding of the intestinal epithelium, crypt dilatation, reactive inflammatory changes.
Grem1	Breast, colorectal.	Proliferation, motility, invasion, ectopic crypt formation.
Chordin	Cervical, gastric, lung, renal, urothelial.	Reduced survival.
Chordin‐like1	Breast, gastric, glioblastoma, lung, leukaemia, melanoma.	Reduced migration and invasion.
Chordin‐like2	Breast, colorectal, glioblastoma, lung.	Proliferation, migration, invasion, chemotherapy resistance.
Follistatin	Basal cell carcinoma, breast, colorectal, gastric, gonadal, hepatocellular carcinoma, lung, melanoma.	Migration, invasion, metastatic formation.
Sclerostin	Breast, colorectal.	Invasion, metastatic formation.

### Noggin

Noggin, also known as Nog and encoded by the *NOG* gene, is a BMP antagonist that is highly homologous between human, rat, and mouse and has therefore earned its place as a potent BMP antagonist that is widely used in cell and organoid culture systems to inhibit BMP signalling. Noggin was first discovered in *Xenopus* due to its ability to restore normal dorsal–ventral body axis patterning in embryos [43], and has since gained significance in its ability to form germ layer‐specific derivation of specialized cells [44].

The function of Noggin as a BMP inhibitor is well established, with significant implications in the context of CRC progression. Noggin can act to maintain intestinal stem cells, acting in parallel with WNT activation to release PTEN inactivation of β‐catenin and therefore target WNT signalling to activated intestinal stem cells [45]. Treatment with Noggin increases the levels of inactive phosphorylated PTEN and active phosphorylated AKT oncogenic pathways [45]. Abnormal branching and budding of the intestinal epithelium occurs during Noggin overexpression, with resulting crypt dilatation and reactive inflammatory changes in the epithelium [46].

**Figure 4:**
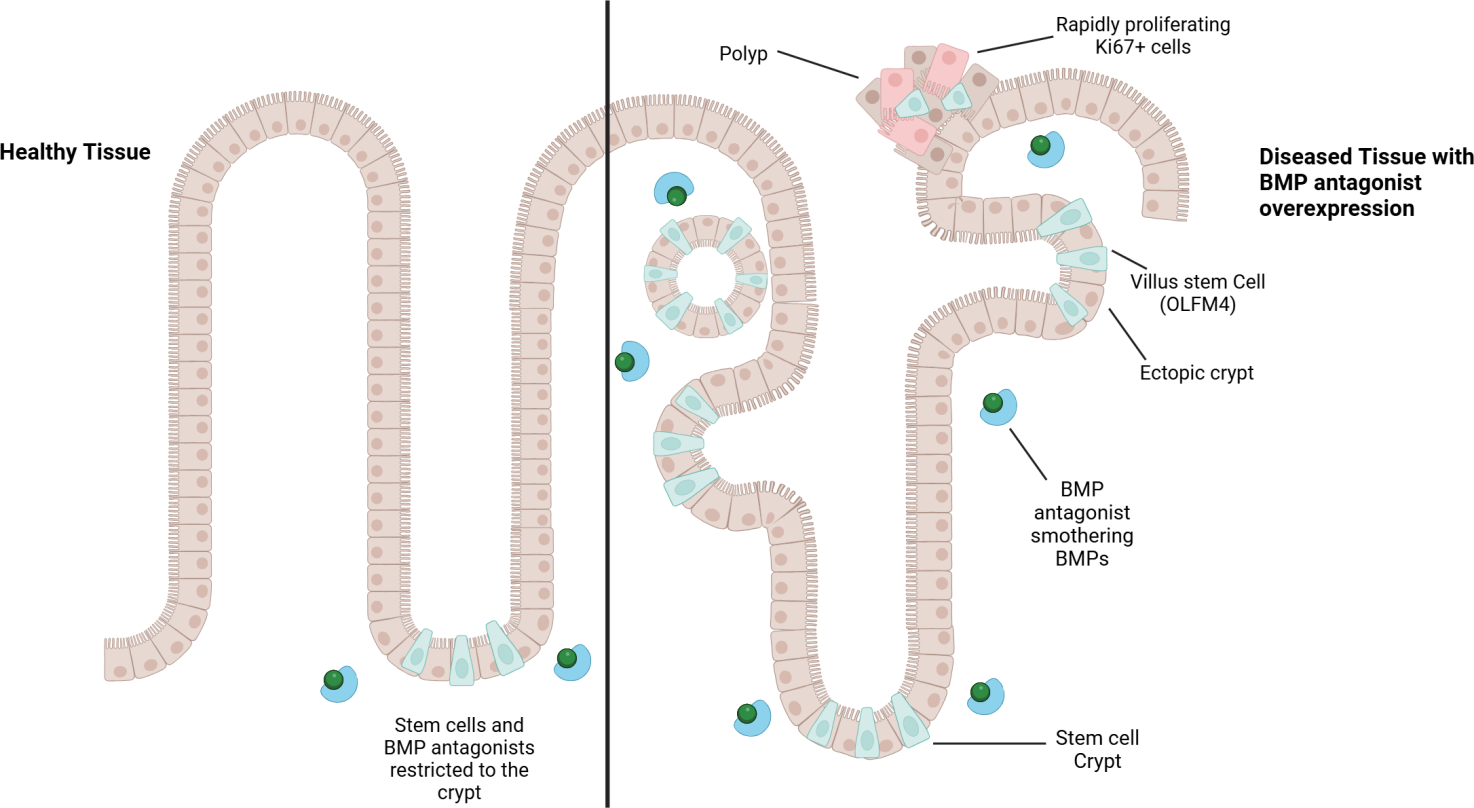
Ectopic crypt and polyp formation following BMP antagonism by Noggin and GREM1. Created in BioRender. Lewis, A. (2025) https://BioRender.com/p16o696. All terms of the License Terms including all Prohibited Uses are fully complied with.

Due to the tumour suppressive nature of BMP signalling, Noggin has been implicated in a wide variety of cancers, including breast, gastric, colorectal, and skin tumorigenesis [42, 47]. BMP inhibition by Noggin in transgenic mice resulted in phenotypic similarities to the human condition Juvenile‐polyposis syndrome, which is characterised by BMP dysregulation. This includes the formation of numerous ectopic crypts perpendicular to the crypt–villus axis characterised by proliferating Ki‐67^+^ cells, increased β‐catenin, and polyp development (Figure 4) [48]. Noggin has also proven vital for culturing human and murine intestinal organoids [49, 50]. Noggin is used to antagonise differentiation mediated by BMP signalling cues in organoid cultures, allowing maintenance of stem‐cell populations that sustain organoid growth and replantation.

Despite these observations that Noggin enhances stem‐cell characteristics in organoids and transgenic mice, useful but artificial research models, there is less conclusive evidence for the role in human CRC patients. Noggin is low in normal and cancer tissues, and mutations and amplifications are rare [51, 52, 53]. Within CRC, Noggin is not an independent prognostic factor [54], although patients with low noggin levels have increased survival rates, especially in combination with high expression of the BMP target gene, *ID1* [54]. These inconsistencies around the role of Noggin could in part be due to the tissue‐ and cell type‐specificity of Noggin expression. Mutations and expression changes of Noggin, and of other BMP antagonists, are more likely to be biologically important in the mesenchymal cells of stromal tissue rather than the epithelial cells.

### Gremlin 1

Gremlin1 (encoded by *GREM1*), previously known as Drm, is a 20kDa highly conserved glycoprotein that acts as a BMP antagonist. Gremlin1 is part of the DAN family of proteins, which is a subgroup of the CAN (Cerberus and dan) family and is a secreted glycosylated protein that contains a C‐terminal cysteine knot with an eight‐membered ring [55, 56]. Gremlin1 was originally identified as a pathogenic mediator of diabetic nephropathy [57], and has a variety of functions as a BMP antagonist, including the regulation of limb bud formation [55, 58]. In CRC, high *GREM1* mRNA levels associate with poor patient outcome. Moreover, Gremlin1 neutralising antibodies were able to shrink tumour size and promote Lgr5^+^ intestinal stem‐cell differentiation, confirming that Gremlin1 can be oncogenic *via* promoting WNT stem cell signalling [40]. Furthermore, BMP antagonism through Gremlin1 causes regenerative stem‐cell activation, suggesting that BMP antagonism is able to functionally switch cells into a dedifferentiated state [59]. Overexpression of *GREM1* was also shown to enhance the motility and invasion of CRC cells by epithelial–mesenchymal transition (EMT), as well as upregulating activating transcription factor 6 (ATF6) and downregulating ATF4, modulating the unfolded protein response through activation of PI3K/AKT/mTOR and antagonizing BMP2 signalling pathways [60].

One of the most significant roles of Gremlin1 in disease initiation is its role in hereditary mixed polyposis syndrome (HMPS), a highly penetrant disorder predisposing to CRC. A 2012 study found that HMPS was caused by a germline gene duplication spanning the 3’ end of the *SCG5* gene and a region upstream of the *GREM1* locus [16]. In the intestine, Gremlin1, as with most BMP antagonists, is largely restricted to mesenchymal cells. However, this mutation caused ectopic expression of *GREM1* mRNA in epithelial cells and concurrent loss of BMP signalling. This is supported by finding a similar duplication upstream of the *GREM1* gene in a family with attenuated/atypical polyposis syndrome [61]. HMPS polyps are characterised by ectopic crypts that develop orthogonally to the crypt axis and contain actively proliferating cells. Davis *et al* modelled the HMPS *GREM1* duplication by using a transgenic mouse line, *Vil1/Grem1*, that overexpress *Grem1* specifically in intestinal epithelial cells [62]. The intestines of these mice developed ectopic crypts, in a similar fashion to HMPS patients, which budded to become actively proliferating intravillus lesions, causing pan‐intestinal polyposis with mixed serrated, adenomatous, and cystic phenotypes (Figure 4). Reduction in phosphorylated SMAD1/5/8 confirmed that *Grem1* overexpression disrupted the BMP signalling gradient in the vertical axis on the intestinal villi, causing the intestines of *Vil1/Grem1* mice to be 28% longer than their wildtype counterparts.

### Chordin family

The Chordin family of proteins are BMP antagonists with highly conserved Chordin‐like cysteine‐rich domains [63, 64]. Three proteins in this family have been identified: Chordin, Chordin‐like 1, and Chordin‐like 2 [38]. Chordin acts as a BMP antagonist through binding to BMPs to prevent heterodimerisation of BMP dimers to BMPRs I and II. This process is reversible, and Chordin can be cleaved from BMPs by the metalloproteinase, Tolloid [65]. A further level of regulation comes from Sizzled, which acts as a negative inhibitor for BMP signalling by inhibiting Tolloid [65].

### Chordin

Chordin (encoded by *CHRD*) has been identified as a negative prognostic factor in CRC and several other cancers, including lung, cervical, urothelial, renal, and stomach cancers, and is correlated with significantly lower survivability [64, 66]. Over 70% of multiple cancer types including lung squamous cell carcinoma, uterine carcinoma, and oesophageal carcinoma patients have increased *CHRD* copy number variants (CNVs) [64]. This suggests that Chordin is a negative prognostic biomarker, acting to decrease life expectancy. However, there is no current published data on the mechanistic role of Chordin in CRC [6].

### Chordin‐like 1

As with Chordin, Chordin‐like 1 (encoded by *CHRDL1*) is also a negative prognostic marker in several cancer types. *CHRDL1* was previously shown to be expressed in colon crypts, and to act to maintain the colonic epithelial stem cell niche through modulation of WNT activity by BMP inhibition [6, 67]. However, single‐cell RNA sequencing (scRNAseq) data shows a lack of *CHRDL1* mRNA in normal colon cell types [52]. It is possible that more contaminating myofibroblasts and nonepithelial cells were present in the dissected crypts [6] than in the scRNAseq data, from which they can be easily excluded. Moreover, a recent study of bulk RNAseq data from TCGA colon adenocarcinoma (COAD) samples shows a reduction in *CHRLD1* mRNA in tumour tissues, potentially due to promoter methylation [68]. Increased *CHRDL1* expression in CRC cell lines has a tumour‐suppressive effect, which was emulated in xenograft models [39, 69, 70, 71].

### Chordin‐like 2

Chordin‐like 2 (*CHRDL2*) was first discovered in 2003 through differential analysis of cDNA fragments in vascularized breast tumours [72]. First termed BNF‐1, *CHRDL2* is overexpressed in breast, lung, and colon tumours [63, 72]. Chordin‐like 2 is a BMP ligand antagonist and binds to BMPs to prevent them from interacting with their cognate cell surface receptors [41, 73]. The *CHRDL2* gene is 35‐kbp and encodes a 47‐kDa protein with a repeated cysteine‐rich motif known as Von Willebrand factor C (VWC) and a signal peptide [63]. The VWC domain mediates the binding of Chordin‐like 2 to BMP2 and 4, as well as to Twsg (twisted gastrulation), allowing the formation of a tertiary complex consisting of BMP, Chordin‐like 2, and Twsg [74]. This tertiary complex allows increased binding affinity of Chordin‐like 2 to BMPs, enhancing its inhibitory effects.

*CHRDL2* mRNA undergoes extensive and complex alternative splicing in different tissue types, with alternative isoforms possibly affecting BMP binding affinity [63]. Chordin‐like 2, like the other family members, can be intracellular or secreted into the extracellular matrix [75]. In the context of CRC, Chordin‐like 2 is upregulated in tumour tissues, and overexpression predicts poor prognosis [76]. Furthermore, high *CHRDL2* levels correlate with clinical features, including tumour size, TNM staging, and tumour differentiation [76]. Genetically predicted Chordin‐like 2 protein levels were also shown to correlate with increased CRC risk [77]. Coimmunoprecipitation assays have shown that Chordin‐like 2 binds to BMP2, 4, and 6. Chordin‐like 2 antagonism of BMPs blocks SMAD1/5 phosphorylation, thereby promoting CRC cell proliferation and inhibiting apoptosis. Furthermore, Chordin‐like 2 prevented BMP‐mediated cell cycle arrest, thereby promoting cell cycle progression and CRC cell proliferation *in vitro* and *in vivo* through upregulation of Cyclin D and downregulation of p21^CDKN1A^ [76]. *In vivo* xenografted tumours from *CHRDL2* overexpressing cells displayed increased weight and volume, with increased proliferation shown by Ki67 staining. In addition, *CHRDL2* overexpression in CRC cell lines increased their resistance to chemotherapy by activating DNA repair pathways accompanied by a general enhancement of CSC characteristics due to reduced BMP and elevated WNT signalling [78].

Chordin‐like 2 has also been shown to play a role in other cancer types [79]. In gastric cancer, for instance, *CHRDL2* correlates with a later cancer stage and poor prognosis [80]. Interestingly, *CHRDL2* overexpression in gastric cancer cell lines highlighted misregulation in the YAP/TAZ pathway, which is a potential method by which Chordin‐like 2 could affect gene regulation via WNT activation. The YAP/TAZ pathway consists of transcriptional activators elevated in many cancers that correlate with CSCs, chemotherapy resistance, and loss of contact inhibition [81, 82, 83]. There is also evidence that WNT signalling directly stimulates YAP, as YAP is a target for degradation by the β‐catenin destruction complex [84].

Interestingly, investigations into sporadic CRC using genomewide association studies highlighted several single nucleotide polymorphisms (SNPs) in the *CHRDL2* genomic region associated with increased risk of CRC [17]. Whether these SNPs enhance CRC risk by affecting alternative splicing of *CHRDL2* mRNA or by increasing *CHRDL2* expression *via* epigenetic or gene regulatory mechanisms is yet to be elucidated. However, it is clear that, as a BMP antagonist, Chordin‐like 2 has a definitive role in the inhibition of BMP and promotes tumourigenicity.

### Follistatin

Follistatin (encoded by *FST*) is a secreted glycoprotein and an endogenous neutralizer of BMPs. Follistatin is a well‐documented inhibitor of TGF‐β ligands, and binds in a 2:1 stoichiometry, occluding BMPR type I and II binding domains [85]. Follistatin is abundantly expressed in several solid tumours, such as breast, gonadal, gastric, lung, and CRC, as well as hepatocellular carcinoma, basal cell carcinoma, and melanoma [86, 87], enhancing cell proliferation, metastases, and angiogenic factors, and acts primarily in a protumourigenic fashion [85]. In CRC, elevated Follistatin‐like 3 (*FSTL3*) promotes migration, invasion, and metastatic formation by directly activating β‐Catenin‐mediated EMT and aerobic glycolysis. In mouse xenograft models, *FSTL3* was linked to increased metastatic formation of CRC cells [86, 88, 89].

### Sclerostin

Sclerostin, a secreted glycoprotein encoded by the *SOST* gene, is a BMP antagonist, primarily produced by osteocytes and with roles in bone formation. Sclerostin has some homology to the DAN family of BMP antagonists and more distantly to Noggin [90]. However, its function in cancer is not straightforward, as it may inhibit both BMP and WNT signalling. Originally identified as a BMP antagonist, Sclerostin competes with BMPRI and II for binding with BMPs 4, 5, and 6, blocking subsequent SMAD phosphorylation and leading to decreased BMP signalling [91]. However, Sclerostin's binding affinity to BMPs is weak compared to other DAN family members, and new research has found that its primary role may be in directly inhibiting the WNT signalling pathway [92, 93, 94].

## BMP inhibitor complexes

Several proteins have also been identified that may promote the activity of BMP antagonists through complexing with BMPs and enhancing antagonist binding affinity. These proteins, such as Twisted gastrulation protein‐1 (Twsg1, encoded by *TWSG1*) and Crossveinless (*CV*), enhance BMP inhibition and may promote cancer formation. Twsg1 is an evolutionarily conserved secreted glycoprotein that binds to Chordin and directly to BMPs 2 and 7, facilitating the formation of a Chordin‐BMP‐Twsg1 complex [95]. The trimolecular complex prevents BMP from binding to its receptors, thus elevating the efficacy of Chordin's BMP antagonist role [96, 97]. In papillary thyroid cancer, *TWSG1* knockdown inhibited migration, invasion, and proliferation [98], suggesting a role in enhancing cancer cell functionality. However, the role it may play in CRC, where expression is low [97], is yet to be elucidated.

Crossveinless (also called BMP‐binding endothelial regulator, encoded by *BMPER*) is a Twsg1‐like protein that also interacts with Chordin family members to modulate BMP signalling [99]. Despite this common property, Crossveinless shows a different BMP ligand specificity than Twsg. Crossveinless can be separated into two subclasses, 1 and 2, and as with other BMP antagonists, is a secreted protein that inhibits BMP2, 4, and 6. Like Chordin, Crossveinless contains four VWC domains, which interact with BMP cognate cell surface receptors. Crossveinless promotes angiogenesis, and is highly expressed in CRC, lung, and cervical cancers, associating with tumour angiogenesis and malignancy [77, 100].

## CRC cell‐of‐origin and the role of BMP antagonists

There has long been debate as to whether CRC is formed by intestinal stem cells (known as the bottom‐up model), or by activation of differentiated cells further up the villi (known as the top‐down model). The bottom‐up model is frequently illustrated by knockout mutations of the *Apc* gene in mice, which results in activation of WNT signalling and the formation of microadenomas. *Apc* knockout is necessary in LGR5^+^ cells in the intestinal crypt for tumour formation and few effects are seen with knockout in committed lineages higher up in the villus or in the transit‐amplifying zone, where microadenomas rapidly stall [101, 102]. The top‐down model proposes that insult to differentiated epithelial cells in the intercryptal zone results in a stem‐like transformation, possibly due to activation of inflammatory pathways such as NF‐κB, leading to the lateral spread of cancer cells that fill the intestinal crypt [103, 104]. In cases of chronic inflammation, perhaps caused by Western‐diet habits, NF‐κB modulates WNT signalling to induce dedifferentiation of nonstem cells that then acquire tumour‐initiating capacity. Most recently, studies have shown that differentiated Paneth cells, upon inflammatory‐associated *APC* loss or targeted mutation of *APC*, initiate tumour formation in patients with inflammatory bowel disease and in sporadic colon cancer [105]. Thus, a top‐down cascade is proposed in which the tumour does not originate from the intestinal stem cell [103].

Current understanding supports both these models [106, 107], and suggests that the cell‐of‐origin is in fact due to the nature of the disruption of the WNT/BMP counter‐gradient, which can be modulated by additional factors affecting cellular plasticity, including injury, inflammation, and related signalling pathways such as YAP/TAZ, Notch, and NF‐κB. If WNT is activated directly, the stem cell compartment is expanded, with tumours more likely to initiate from LGR5^+^ cells [101]. However, if cellular plasticity is increased by direct BMP repression, for example, due to *SMAD4* loss or *GREM1* activation, or by indirect BMP repression due to injury/inflammation, cells outside the crypt base/stem cell compartment can dedifferentiate into stem cell precursors [28, 62].

## Targeting BMP in cancer

The potential for targeting the BMP signalling pathway for CRC treatment is clear, and a number of studies have begun to explore this. The use of BMPs to counteract aberrant WNT signalling has shown promising results, with BMP4 addition shown to induce differentiation and apoptosis of CRC CSCs. Furthermore, BMP4 enhances the cytotoxic effects of the chemotherapy drugs 5‐FU and oxaliplatin [27]. BMP2 has also been identified and used as a differentiating and radiosensitising agent for CSCs [108]. BMP2 treatment significantly reduced stem cell markers, EMT‐related genes, and DNA repair pathway proteins, suggesting that restoring the BMP signalling may provide a novel therapeutic treatment for CRC. Furthermore, treatment with a stabilized BMP7 variant mutated to reduce BMP antagonist interactions enhanced therapeutic sensitivity, suggesting that modified BMPs have therapeutic potential [34].

Mouse models of autocrine BMP4 ligands show reduced proliferation, increased terminal differentiation, abrogated secretory cell survival, and lack of dedifferentiation [59]. However, these effects are reversed through stromal upregulation of *GREM1*, with attenuation of the BMP signal, allowing intestinal regeneration. Therefore, BMP treatment may not be sufficient in cases of BMP antagonist overexpression, and BMP antagonist targeting would be required. Indeed, the use of antibodies to target BMP antagonists has also been investigated. Recent attention to the use of an anti‐Gremlin 1 antibody in various diseases has been discovered as a potential therapeutic target. Initially, a Gremlin 1 antibody that prevents Gremlin 1‐BMP interaction was developed by Novartis (Indianapolis, IN, USA) for pulmonary artery hypertension [109]. Another study suggested that anti‐Gremlin 1 could decrease A549 lung cancer growth through reduction in cellular proliferation, invasion, and migration [110]. In CRC, a Gremlin 1 human IgG4p monoclonal antibody, Ginisortamab, has recently been developed by UCB (Brussels, Belgium) and has been shown to neutralise Gremlin 1 antagonism of BMP2 and to have antitumour effects in a preclinical murine trial [40]. Transcenta have also developed a monoclonal Gremlin 1 antibody (TST003) that inhibits Gremlin 1‐mediated noncanonical activation of FGFR1 phosphorylation [111]. It is clear that targeting Gremlin 1 has produced exciting results, and the potential for cancer therapy, especially in patients with HMPS, is evident. In this respect, a recent study [112] suggested that the mixed polyposis phenotype associated with *GREM1* overexpression in HMPS patients could be reversed using a Gremlin 1 antibody. *Vil1/Grem1* mice were produced, which displayed classical features of HMPS, including pronounced pan‐intestinal polyposis and lesions that phenocopy the histology of human patients, including the formation of ectopic crypts [112]. After treatment with antibody (UCB Ab7326), mice showed a reversion of the characteristic *Vil1/Grem1* pan‐intestinal polyposis phenotype with an increase in mean survival. Gremlin 1 antibody treatment led to rapid abolition of polyposis and near elimination of ectopic crypts, with markers of stemness and proliferation restricted to the crypt [112]. These data support previous finings that aberrant *GREM1* expression induces an intestinal architectural change to exhibit a mixed polyposis phenotype, and that this is a reversible *via* Gremlin 1‐targeting antibodies. This suggests that BMP antagonist‐mediated polyposis can be both prevented and reversed through sequestering inhibition specific antagonists, such as Gremlin 1.

Together, these studies suggest a potential opportunity to target the BMP pathway, be it through BMP treatment or through targeted inhibition of BMP antagonists. This would result in reduced WNT‐driven CSC pools, and an enhanced response to chemo‐ and radiotherapy.

## Future perspectives

There is now overwhelming evidence that disrupting the BMP gradient has profound effects on CRC initiation and progression due to disruption of the opposing WNT morphogenetic gradient. Disruption of the BMP/WNT gradient can be further amplified by the production of BMP antagonists, whose overexpression results in reduction of differentiation‐related BMP signalling and the dedifferentiation of epithelial cells. This manifests in accumulation of CRC CSC pools, which drive tumour initiation, progression, and survival upon chemo‐ and radiotherapy treatment. Furthermore, BMP antagonist overexpression results in a general deregulation of intestinal homeostasis, so much so that ectopic crypt formation and neoplastic lesions can be observed without accompanying WNT pathway mutations.

It is clear, therefore, that BMP antagonists are an important potential therapeutic target for CRC. Indeed, treatment with BMPs has induced differentiation and reduces proliferation of cancer cell lines, and through targeting BMP antagonists we can disrupt tumourigenic effects. Anti‐Gremlin 1 antibodies are currently being explored for CRC treatment [40, 112], which could prove especially influential in patients with increased antagonist levels, such as those with HMPS caused by ectopic *GREM1* expression. There is clearly potential for the use of these and antibodies for other BMP antagonists such as Chordin‐like 2 in sporadic CRC, where increased levels of several BMP antagonists correlate with poor patient survival.

In addition to the therapeutic potential of BMP antagonists, their role as biomarkers of prognosis and to predict response to chemotherapies should also be explored further. In CRC, there is a clear correlation of Chordin‐like 2 with survival and stage, and both Gremlin 1 and Chordin‐like 2 have been identified as plasma biomarkers of CRC [77]. The resistance to chemotherapy seen with high levels of BMP antagonists reflects the relationship between cancer cell stemness and enhanced DNA repair. This merits further research into the use of BMP antagonists to predict therapeutic response, as well as explorations of targeted BMP antagonist inhibition in combination with standard chemotherapy regimens.

## Conclusion

Maintaining the balance of BMP and WNT signalling in the intestine is key to homeostasis and suppressing cancer development. This can be disrupted by excess BMP antagonist expression, which blocks BMP signalling and differentiation, resulting in increased populations of intestinal stem cells. These cells generally have an increased capacity for proliferation and migration, and exhibit chemo‐ and radiation‐resistance. However, there is conflicting evidence if this would increase proliferation and metastasis, and tissue type and context are important. Upregulation of Gremlin 1, Noggin, or Chordin‐like 2, is protumorigenic in CRC. However, other BMP antagonists may reduce BMP‐mediated hyperproliferation in CRC and other cancer types.

In summary, BMP inhibition has been shown to be a crucial component of deregulation in intestinal homeostasis and a vital part of CRC development. It is clear that research into BMP antagonism inhibition offers exciting opportunities for CRC treatment.

## Author contributions statement

AL and EC conceived and wrote the article.

## Data Availability

Data sharing is not applicable to this article, as no new data were created or analysed in this study.
